# Depression and anxiety in people with kidney disease: understanding symptom variability, patient experience and preferences for mental health support

**DOI:** 10.1007/s40620-024-02194-1

**Published:** 2025-01-12

**Authors:** Joseph Chilcot, Christina J. Pearce, Natalie Hall, Zara Rehman, Sam Norton, Sophie Griffiths, Joanna L. Hudson, Lucy Mackintosh, Amanda Busby, David Wellsted, Julia Jones, Shivani Sharma, Paula Ormandy, Nick Palmer, Pooja Schmill, Maria Da Silva-Gane, Neal Morgan, Dimitrios Poulikakos, Kristin Veighey, Stuart Robertson, Rob Elias, Ken Farrington

**Affiliations:** 1 Department of Psychology, Institute of Psychiatry, Psychology and Neuroscience, King’s College London, 5th Floor Bermondsey Wing, Guy’s Campus, London Bridge, London, SE1 9RT UK; 2 School of Life and Medical Sciences, University of Hertfordshire, College Lane Campus, Hatfield, UK; 3 School of Health and Social Work, University of Hertfordshire, Hatfield, UK; 4 Aston Business School, Aston University, Birmingham, UK; 5 School of Health and Society, University of Salford, Salford, UK; 6 Kidney Care UK, Alton, UK; 7 Renal Unit, Lister Hospital, Stevenage, UK; 8 Daisy Hill Hospital, Southern Health and Social Care Trust, Craigavon, Northern Ireland UK; 9 Salford Royal Hospital, Northern Care Alliance NHS Foundation Trust, Salford, UK; 10 University Hospital Southampton, NHS Foundation Trust, Southampton, UK; 11 Wrexham Maelor Hospital, Wales, UK; 12 King’s College Hospital, NHS Foundation Trust, London, UK

**Keywords:** Depression, Anxiety, Mental health, Chronic kidney disease

## Abstract

**Background:**

Depression and anxiety are commonly experienced by people with chronic kidney disease (CKD). This study aimed to evaluate person- and service-level factors associated with depression and anxiety symptoms. We sought to also understand utilisation of mental health treatments and preferences for future psychological support.

**Methods:**

An online survey recruited participants from six UK kidney services with varying levels of psychosocial provision. The survey was also advertised on social media. Participants completed screening questionnaires for depression and anxiety, alongside questions about mental health history, self-efficacy, treatment and support. The study included adults (18 years or older) living with CKD (stages 3b and above) or those receiving any form of Kidney Replacement Therapy (KRT), including individuals with a functioning kidney transplant. Eligible participants had to complete study measures and be proficient in reading and writing in either English or Welsh, as the survey was administered in these languages. This survey was developed with our Patient and Public Involvement group and was administered from January 2023 until 31st January, 2024 using Qualtrics and RedCap.

**Results:**

Four hundred fifty-eight people completed the survey. Moderate-severe symptoms of depression and anxiety were 37.7% and 26.5%, respectively. Over 50% reported a history of diagnosed depression. In addition to depression, sleep problems and fatigue were identified as future support needs, with over a third indicating a preference for in-centre provision. In case-mix adjusted analysis, there was no variability in depression and anxiety symptoms across centres. Centre location and size were unrelated to symptoms. Age, female gender, current mental health treatments, self-efficacy and perceptions regarding opportunity for support, were associated with symptoms of depression and anxiety. In sub-analysis, there was a negative association between psychosocial staffing levels and depression symptoms.

**Conclusion:**

Patient-related factors and behavioural characteristics were related to variation of these symptoms. There was little evidence of symptom variability across centres, although in a small sub-analysis, psychosocial provision showed a weak negative correlation with depression symptoms. Our findings highlight preferences of future needs which could be helpful for designing future research and service provision.

**Graphical abstract:**

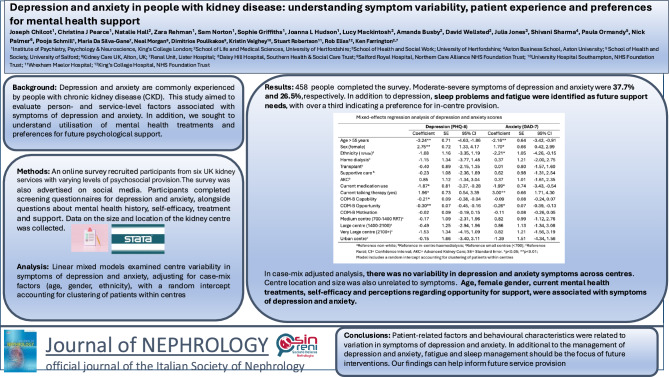

**Supplementary Information:**

The online version contains supplementary material available at 10.1007/s40620-024-02194-1.

## Background

Psychological distress is common in people living with chronic kidney disease (CKD) [1]. Depending on the assessment methods undertaken, between 22–39% and 19–43% of CKD patients experience depression and anxiety, respectively [2, 3]. Across the spectrum of CKD, there is evidence of poorer clinical outcomes in people with depression and anxiety, including increased disease progression and higher mortality [4, 5]. Therefore, understanding and improving mental health in CKD is crucial for person-centred care.

Despite the prevalence of depression and anxiety in this setting, management options are limited. There is a lack of robust evidence concerning the effectiveness of antidepressant medications [6, 7], though they are frequently prescribed, usually in primary care, and poorly monitored [8]. There is more evidence for talking therapies [9], though significant logistical problems exist in providing access to these treatments. There is evidence of variable psychosocial practice patterns in kidney care, notably staffing ratios of psychosocial services, contributing to mental health disparities [10–12]. There is specific evidence showing that higher psychosocial staffing is associated with less psychological distress across UK kidney centres [10]. Furthermore, there are greater patient-reported unmet needs in centres with low or no psychosocial staffing provisions [10].

Another major factor in tackling these issues is the perception that people living with advanced CKD have regarding psychosocial provision, including how, when and indeed whether to access it. Whilst there has been a growing body of research examining biopsychosocial factors associated with mental health in CKD, there is little work on help-seeking behaviour or perceptions regarding access to support. Help-seeking for mental health refers to a coping process where individuals seek to obtain formal and informal support when psychological distress is experienced [13]. Whilst service availability remains a significant barrier to help-seeking, other factors include stigma, functional impairment, self-efficacy and illness beliefs [14, 15].

To further understand perceptions regarding access to mental health support in people with CKD, the present study utilised the Capability, Opportunity, and Motivation (COM-B) framework of health behaviour [16]. This framework consists of three components (capability, opportunity, and motivation) that can influence whether an individual engages in a behaviour, such as help-seeking and accessing treatment. Capability includes both the physical and psychological skills that are necessary in performing a behaviour. Opportunity relates to both the physical (e.g., adequate time and resources) and social (e.g., cultural norms, and interpersonal influences) opportunity to engage in a behaviour. Motivation includes reflective (conscious planning to perform the behaviour) and automatic motivation (performing the behaviour without conscious planning). In the context of CKD, COM-B framework has been applied to understand barriers and facilitators of exercise [17], and used to inform behaviour change interventions [18, 19].

This study aimed to evaluate person- and service-level factors associated with symptoms of depression and anxiety. These factors included the size and location of renal centres, potentially indicative of varying levels of psychosocial service provision, along with patient demographics, clinical characteristics and psychological factors. In addition, we sought to understand patients' utilisation of mental health treatments, providing insights into medication experiences, psychological support receipt, and preferences for future psychological support.

## Methods

### Study design

This online survey recruited participants from six UK renal services with varying levels of psychosocial provision identified from past research for inclusion in a mixed-methods study [20]. Since recruitment was lower than anticipated, the survey was replicated and advertised on social media asking participants to report the centre in which they receive their kidney care. The analysis here reports data from both the clinic and online samples. Ethical approval was granted from the UK Health Research Authority and a local university committee.

### Participants

The study included adults (18 years or older) living with CKD (stages 3b and above) or those receiving any form of Kidney Replacement Therapy (KRT), including individuals with a functioning kidney transplant. Eligible participants had to complete study measures and be proficient in reading and writing in either English or Welsh, as the survey was administered in these languages. Participants were ineligible if they had a known cognitive or learning impairment, psychosis, and if they lacked English or Welsh language proficiency.

### Procedure

This survey was developed with our Patient and Public Involvement group and was administered from January 2023 until 31st January, 2024 using Qualtrics and RedCap. Convenience sampling was used to recruit the online sample through advertisements circulated across social media channels. Interested individuals were directed to a study information webpage. Electronic informed consent was obtained, and eligible participants proceeded to complete the survey, requiring approximately 15–20 mins of their time. The clinic sample recruited individuals through advertisement on patient clinical letters and in-person interactions with their medical care teams. Researchers attending clinics were introduced to potential participants by the medical team. Participants choosing to complete the questionnaire during clinic visits were guided through the online information sheets, consent form and questionnaire. Information sheets outlined additional support details, and the consent form sought permission to refer participants for help if depression screening scores indicated high depressive symptoms.

### Measures

#### Demographic and clinical variables

Participants provided information on demographics and clinical aspects including age, gender, ethnicity (categorised as per the Office for National Statistics 2021 guidelines), CKD stage/modality and primary kidney disease diagnosis. In the online sample, participants selected their primary renal centre from a dropdown list containing the names of the centres. Self-reported clinical data included the primary cause of CKD, current treatment or nature of disease management, and co-morbidity evaluated by a validated self-report tool[21].

#### Psychological wellbeing

Participants provided self-reported information on both historical and current mental health conditions, including details such as age at diagnosis, the diagnosing professional, and healthcare professionals consulted about their mental health. Symptoms of anxiety and depression were measured using the Generalised Anxiety-7 questionnaire (GAD-7)[22] and the 8-item version of the Physical Health Questionnaire (PHQ-8)[23].

The GAD-7 measures general anxiety for the preceding two weeks through seven Likert-scale items, scored from (0), not at all, to (3), nearly every day. Higher scores, ranging from 0 to 21, indicate greater anxiety severity. A cut-off score of ≥ 10 can be applied to indicate potential generalised anxiety disorder. The PHQ-8 is a condensed version of the PHQ-9 which does not include the ninth item regarding suicidal ideation. The PHQ-8 evaluates eight depression-related symptoms over the past two weeks. Each item, scored on a Likert scale from (0), not at all, to (3), nearly every day, contributes to a total score ranging from 0 to 24, with higher scores being indicative of greater depression severity. A PHQ-8 score ≥ 10 indicates potential clinical depression.

#### Treatment and management

Participants' medication and treatment experiences over the previous 12 months were collected. Participants were asked what medications they were taking, selecting from a list of the most frequently prescribed antidepressants, who prescribed them, and whether they are currently taking the medication. Regarding talk therapies, participants were asked about any sessions received from a healthcare professional in the previous two years. Specifically, participants were asked to detail the type of counselling or therapy, the provider, the method of delivery, and the number of sessions attended. Participants were also asked about the use of common complementary, alternative, or other therapies.

#### Perceived access to psychological support and mental health self-efficacy

Participants' experiences in accessing psychological support over the previous 12 months were evaluated using the Capabilities, Opportunities, Motivations (questionnaire) [24]. The six-item questionnaire was recently developed to measure perceived capabilities (physical and psychological), opportunities (physical and social), and motivations (reflective and automatic). The questionnaire features generically phrased questions adaptable to different behavioural goals and, for this study, it specifically explored “access to psychological support” to depict help-seeking for mental health behaviour. Each item is scored on a Likert scale from (0), strongly disagree, to (10), strongly agree, with higher scores indicating greater perception of capabilities, opportunities, and motivations. Self-efficacy related to mental health was measured using Mental Health Self-Efficacy Scale (MHSES)[25]. Comprising six items, this questionnaire measures an individual's confidence in performing behaviours related to mental health self-care. Each item is rated on a Likert scale from (1), Not at all confident, to (10), Totally confident. Scores ranged from 6 to 60 with higher scores indicating greater mental health self-efficacy.

#### Preferences for future psychological support

Participants' future preferences for psychological support were explored—specifically who they would prefer as providers (e.g., the renal team or external healthcare professionals), the types of healthcare professionals or services (e.g., kidney disease charity, faith-based organisation), the desired forms of therapy, including format preferences (e.g., face-to-face, telephone), and the specific topics they wish to address (e.g., depression, maintaining a healthy diet, family planning).

#### Renal centre characteristics

The service-level factors used in this study to represent levels of psychosocial service provision were size and urban–rural classifications of renal centres. Four size classifications for the renal centres were used based on the number of patients receiving KRT in 2021 (UK Renal Registry), small (patient size, < 700), medium (700–1400), large (1400–2000) and very large (2100+). The urban–rural classification of renal centres was determined by considering the population density per square mile of the city where each renal centre was located. This classification involved utilising the median split of the population density per square mile to dichotomise the variable, designating centres with a density of ≤ 251 as rural and those with a density > 251 as urban.

### Sample size

The intended sample size for the clinic sample was 100 patients per site, totalling 600 patients, which was estimated on the basis of providing 80% power at the 5% significance to detect at least small variability (*d* = 0.15) in levels of depression between centres using an *F*-test. It became apparent that this sample size was not achievable. Recruitment was supplemented by an online survey where the target total sample size was maintained by reducing the target per recruiting centre, and the aim to compare depression rates between centres directly removed from the analysis plan.

### Data analysis

Data analysis was conducted using Stata version 18.0. The first part of the analysis was descriptive, focusing on the characteristics of the sample and exploring differences between the two samples including treatment preferences. The overarching aim was to describe variations in practice across centres rather than make direct comparisons between centres. Consequently, significance testing was generally limited to assessing the overall variability of centres from the grand mean, rather than pairwise comparisons of centres. For the levels of current mental health symptoms (PHQ-8 and GAD-7), across-centre models were constructed to compare rates across centres, with the centre with the lowest level of symptoms used as the reference. Differences between centres were tested at the Bonferroni corrected 5% significance level.

Linear mixed models examined centre variability with regard to symptoms of depression and anxiety, adjusting for case-mix factors (age, gender, ethnicity, recruitment modality method, centre size and location), with a random intercept accounting for clustering of patients within centres. As such, 95% confidence intervals (CIs) (and *p*-values) were based on standard errors respecting the hierarchical nature of the data. Further linear mixed models were estimated to identify individual and service level factors associated with symptoms of depression and anxiety. Covariates included in these models were age, gender, ethnicity, renal centre characteristics, medication use for mental health, receipt of psychological support and Capability, Opportunity, and Motivation variables. Due to the high correlation and conceptual overlap between self-efficacy and the Capability, Opportunity, and Motivation constructs, self-efficacy was not included in the model. Instead, the models were rerun with self-efficacy included in substitution of the Capability, Opportunity, and Motivation variables. Data on full time equivalent psychosocial resourcing for some centres was available from our recent study [20], thus included in sub-analysis.

## Results

### Patient and centre characteristics

A total of 458 people completed the survey (online sample = 261; clinic sample = 197). A summary of self-reported sociodemographic and clinical data is shown in Table 1 (self-reported physical comorbidity data are shown in supplementary Table 1 and supplementary Fig. 1). A summary of recruitment by UK renal centre size and geography is shown in supplementary Table 2. Fifty-six centres were represented (plus one unknown).

**Table 1:** Sociodemographic and clinical data

Characteristics	Total	Online	Clinic	*p* value
	*N* = 458	*N* = 261	*N* = 197	
Age (years)				
18–25	20 (4.4%)	17 (6.5%)	3 (1.5%)	< 0.001
26–35	64 (14.0%)	54 (20.7%)	10 (5.1%)	
36–45	50 (10.9%)	29 (11.1%)	21 (10.7%)	
46–55	103 (22.5%)	67 (25.7%)	36 (18.3%)	
56–65	118 (25.8%)	68 (26.1%)	50 (25.4)	
66–75	66 (14.4%)	20 (7.7%)	46 (23.4%)	
76–85	33 (7.2%)	5 (1.9%)	28 (14.2%)	
86+	4 (0.9%)	1 (0.4%)	3 (1.5%)	
Gender				
Male	173 (37.8%)	67 (25.7%)	106 (53.8%)	< 0.001
Female	285 (62.2%)	194 (74.3%)	91 (46.2%)	
Ethnicity				
White	414 (90.6%)	244 (93.5%)	170 (86.7%)	0.035
Asian or Asian British	17 (3.7%)	9 (3.4%)	8 (4.1%)	
Black, Black British, Caribbean, or African	15 (3.3%)	3 (1.1%)	12 (6.1%)	
Mixed or multiple ethnic groups	4 (0.9%)	3 (1.1%)	1 (0.5%)	
Other ethnic group	4 (0.9%)	1 (0.4%)	3 (1.5%)	
Prefer not to say	4 (0.7%)	1 (0.4%)	2 (1.0%)	
Primary cause of CKD				
Diabetes	47 (10.4%)	13 (5.0%)	34 (17.8%)	< 0.001
Hypertension	31 (6.9%)	14 (5.4%)	17 (8.9%)	
Glomerulonephritis	30 (6.6%)	20 (7.7%)	10 (5.2%)	
Polycystic kidney disease	140 (31.0%)	111 (42.5%)	29 (15.2%)	
Lupus or vasculitis	5 (1.1%)	3 (1.1%)	2 (1.0%)	
Other	138 (30.5%)	77 (29.5%)	61 (31.9%)	
I do not know	61 (13.5%)	23 (8.8%)	38 (19.9%)	
Treatment modality				
In-centre haemodialysis	106 (23.7%)	40 (15.6%)	66 (34.6%)	< 0.001
Home haemodialysis	15 (3.3%)	11 (4.3%)	4 (2.1%)	
Peritoneal dialysis	25 (5.5%)	8 (3.1%)	17 (8.6%)	
Kidney transplant	162 (36.2%)	115 (44.7%)	47 (24.6%)	
Supportive/conservative care	77 (17.2%)	37 (14.4%)	40 (20.9%)	
Advance kidney care/low clearance kidney care	63 (14.1%)	46 (17.9%)	17 (8.9%)	

*CKD* chronic kidney disease

Although the online sample was less ethnically diverse than the clinic sample, both were predominantly White. The online sample also had a greater representation of people with a primary polycystic kidney disease diagnosis and those with a transplant. Overall, 259 (56.55%) and 186 (40.61%) reported a history of diagnosed depression and anxiety, respectively. This was notably higher in the online sample (supplementary Table 3). One hundred ninety-three participants (42.14%) reported having been diagnosed with a mental health condition in the past 12 months (online 139 [53.6%], clinic 54 [27.41%]). When asked with which health professionals they had discussed their mental health during the previous year, GPs were the most commonly mentioned in both samples (Supplementary Table 4; *n* = 88, 19.21% overall).

#### Use of medications for mental health

A summary of mental health medication use is shown in supplementary file 5. Overall, 35.5% (*n* = 149) reported being on medication for their mental health, which was higher in the online sample compared with the clinic sample (106 [45.7%], vs 43 [22.9%], respectively). Overall, 52 participants (35.6%) reported being prescribed medication during the previous 12 months, with Sertraline being the agent most used (22 of total sample, 4.8%), followed by Citalopram and Fluoxetine, with GPs being the most reported prescriber (supplementary file 5).

#### Talk therapies and preference for psychological support

With regard to talk therapies, 117 (27.9%) participants reported receiving treatment during the previous 24 months, with counselling and cognitive behavioural therapy being the most common therapy used during the previous 12 months (supplementary file 6). Over a third of respondents indicated a preference for psychological support from health professionals based within the kidney care team, although nearly one half did not mind where the support was located (*n* = 189, 46.9%; supplementary file 7). There was preference for a variety of psychological support interventions including access to cognitive behavioural therapy (*n* = 200, 43.7%), peer support (*n* = 192, 41.9%) and stress-management (*n* = 157, 34.3%). There was a strong preference for individual face-to-face interventions. Respondents indicated preference for a variety of support needs including fatigue (*n* = 218, 67.9%), sleep problems (*n* = 172, 59.3%), depression (*n* = 199, 60.9%) and anxiety (*n* = 150, 51.0%). When asked about the delivery of future support needs, respondents indicated various groups and contexts, with high endorsement of support directly from the kidney team (*n* = 249, 74.1%), and mental health specialists (*n* = 208, 63.4%).

### Symptoms of depression and anxiety

Mean depression (PHQ-8) and anxiety (GAD-7) scores are shown in supplementary Table 8, with higher scores reported in the online sample (*p* < 0.01). Moderate-severe symptoms of depression (PHQ-8 ≥ 10) and anxiety (GAD-7 ≥ 10) were 37.7% (95% CI 34.0, 41.0, *n* = 163) and 26.5% (95% CI 22.0, 30.0, *n* = 116), respectively. Both depression and anxiety scores were negatively correlated with capability, opportunity, motivation and mental health self-efficacy (Mental Health Self-Efficacy Scale; supplementary Table 9).

**Fig. 1:**
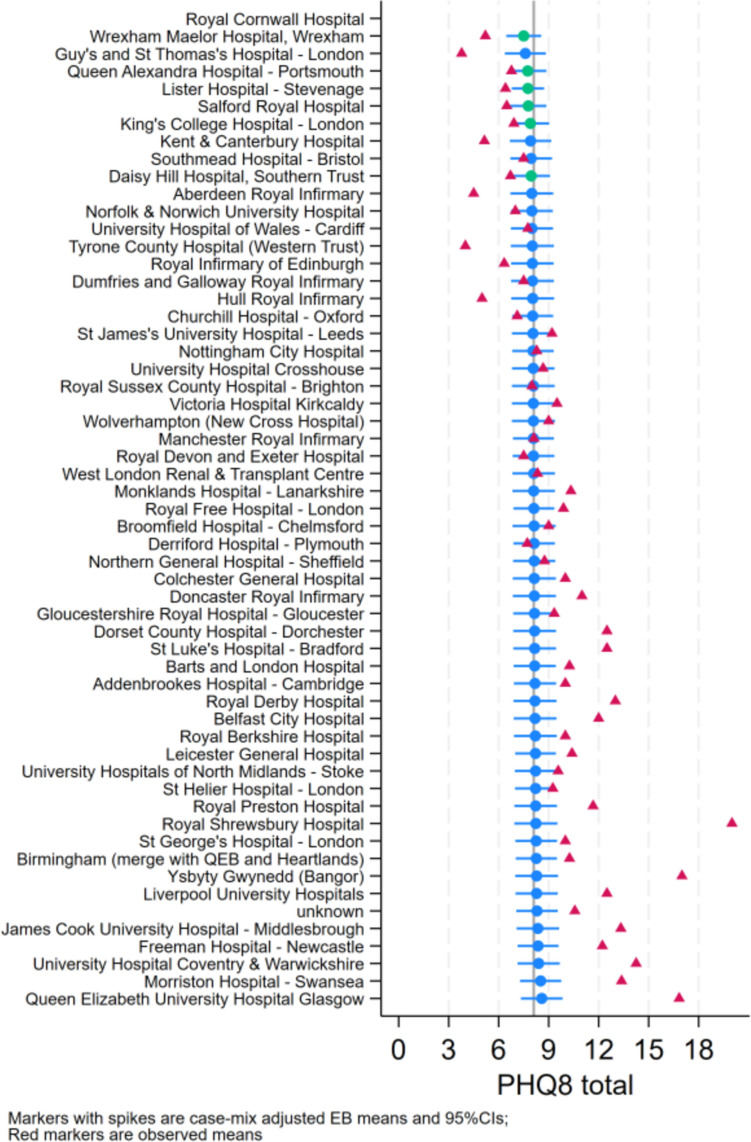
Case-mix adjusted Empirical Bayes (EB) means for depression across centres

**Fig. 2:**
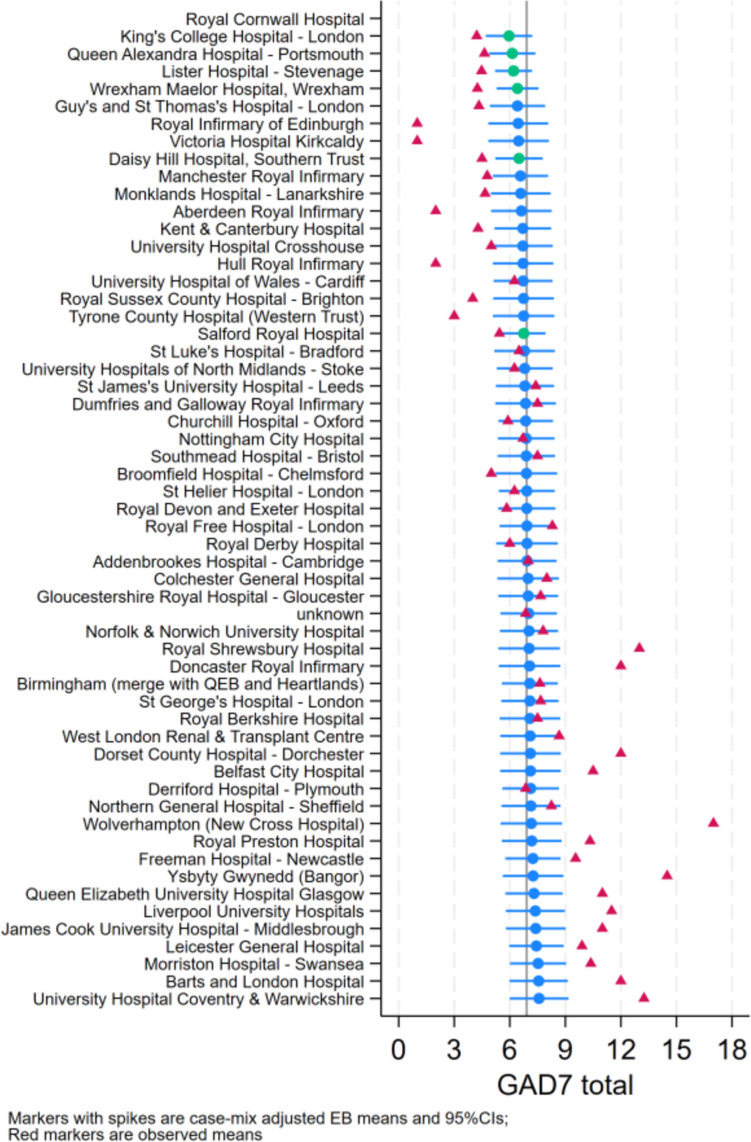
Case-mix adjusted Empirical Bayes (EB) means for anxiety across centres

There was little variability in case-mixed adjusted empirical bayes (EB) means for both symptoms of depression and anxiety across centres (Figs. 1 and 2, respectively). This indicates that variability is largely explained by case-mix rather than centre-level factors. Adjusted mixed effects regression models showed no association between symptoms of depression and anxiety with centre size, location (urban vs. rural) and CKD modality/stage (Table 2). Age had a significant negative relationship with both symptoms of depression and anxiety, which were also higher in females. Anxiety was lower in White individuals than other ethnicity groups. Current use of mental health medication was associated with lower depression and anxiety scores, whereas receiving talk therapy was associated with higher symptoms. Greater perceived capability and motivation was associated with lower symptoms of depression, with opportunity also showing a negative association with symptoms of anxiety. Given the strong relationship between the Capability, Opportunity, and Motivation constructs and mental health self-efficacy, the models were rerun with self-efficacy included and Capability, Opportunity, and Motivation removed. Increased mental health self-efficacy was associated with a decrease in both depression (coeff = − 0.27, 95% CI − 0.30, − 0.23, *p* < 0.01) and anxiety symptoms (coeff = − 0.23, 95% CI − 0.26, − 0.20, *p* < 0.01).

**Table 2:** Mixed-effects regression analysis of depression and anxiety scores

	Depression (PHQ-8)			Anxiety (GAD-7)		
	Coefficient	SE	95% CI	Coefficient	SE	95% CI
Age > 55 years	− 3.24**	0.71	− 4.63, − 1.86	− 2.16**	0.64	− 3.42, − 0.91
Sex (female)	2.75**	0.72	1.33, 4.17	1.70*	0.66	0.42, 2.99
Ethnicity (White)^a^	− 1.08	1.16	− 3.35, 1.19	− 2.21*	1.05	− 4.26, − 0.15
Home dialysis^b^	− 1.15	1.34	− 3.77, 1.48	0.37	1.21	− 2.00, 2.75
Transplant^b^	− 0.40	0.89	− 2.15, 1.35	0.01	0.80	− 1.57, 1.60
Supportive care ^b^	− 0.23	1.08	− 2.36, 1.89	0.62	0.98	− 1.31, 2.54
AKC^b^	0.85	1.12	− 1.34, 3.04	0.37	1.01	− 1.61, 2.35
Current medication use	− 1.87*	0.81	− 3.37, − 0.28	− 1.99*	0.74	− 3.43, − 0.54
Current talking therapy (yes)	1.96*	0.73	0.54, 3.39	3.00**	0.66	1.71, 4.30
COM-B capability	− 0.21*	0.09	− 0.38, − 0.04	− 0.09	0.08	− 0.24, 0.07
COM-B opportunity	− 0.30**	0.07	− 0.45, − 0.16	− 0.26*	0.07	− 0.39, − 0.13
COM-B motivation	− 0.02	0.09	− 0.19, 0.15	− 0.11	0.08	− 0.26, 0.05
Medium centre (700–1400 KRT)^c^	− 0.17	1.09	− 2.31, 1.96	0.82	0.99	− 1.12, 2.76
Large centre (1400–2100)^c^	− 0.49	1.25	− 2.94, 1.96	0.86	1.13	− 1.34, 3.08
Very Large centre (2100+)^c^	− 1.53	1.34	− 4.15, 1.09	0.82	1.21	− 1.56, 3.19
Urban centre^d^	− 0.15	1.66	− 3.40, 3.11	− 1.39	1.51	− 4.34, 1.56

*CI* confidence interval, *AKC* advanced kidney care, *SE* standard error, *KRT* kidney replacement therapy

^a^Reference non-White

^b^Reference in-centre haemodialysis

^c^Reference small centres (< 700)

^d^Reference rural

*p < 0.05; **p < 0.01; model includes a random intercept accounting for clustering of patients within centres

### Impact of psychosocial staffing on depression and anxiety symptoms

In sub-analyses, the adjusted mixed effects regression models were rerun including full time equivalent psychosocial staffing levels (full time equivalent range 0–6.1). Full time equivalent information was available from 20 centres, of which 18 were represented by patients in this survey. Since numbers were relatively small, the psychosocial staffing group was formed by the following professionals: psychologists, psychiatrists, counsellors, trainee/assistant psychologists, social workers and other support workers. Psychosocial full time equivalent had no association with anxiety symptoms but did have a negative association with depression (coeff = − 0.71, 95% CI − 1.33, − 0.09, *p* < 0.05), although the effect was small.

### Association of capability, opportunity, and motivation constructs with centre characteristics

In further sub-analyses, the association between Capability, Opportunity, and Motivation constructs and centre characteristics including full time equivalent was tested using mixed effects regression models, which found no significant associations.

## Discussion

The aims of this study were to evaluate person- and service-level factors associated with symptoms of depression and anxiety to understand patients' utilisation of mental health treatments, providing insights into medication experiences, psychological support receipt, and preferences for future psychological support. In our sample, represented by people across the spectrum of kidney disease, moderate to severe symptoms of anxiety and depression were reported in 26.5% and 37.7%, estimates which align with past studies[2, 3]. The commonality of depression and anxiety across UK kidney care appears uniform, with little evidence of symptom variability across centres when adjusting for case-mix factors. These findings therefore reinforce the need for integrated psychosocial care across all kidney services [10–12].

Approximately half of the sample reported a history of diagnosed depression and anxiety, a third of whom reported medication use for their mental health during the previous 12 months. These self-reported histories are notably higher than previous data [1]. Selective serotonin reuptake inhibitors (SSRIs) were most commonly reported, supporting data from large European studies [26, 27], although there is inconclusive evidence regarding their efficacy in people with CKD [6, 7] and the potential for short-term adverse outcomes [27]. Talking therapies were comparatively less common, despite moderate evidence regarding their efficacy in CKD (e.g. Cognitive Behavioural Therapy) [9]. This largely reflects the chronically under-resourced UK kidney psychosocial work force, despite high demand [12, 20], an issue further highlighted here. Whilst appropriate funding and growth of the psychosocial team remains a significant challenge, over a third of respondents indicated a preference for psychological support from health professionals based within the kidney care team. Although there is a dearth of comparable data in CKD, patient preference for integrated mental and physical health care is reported in other long-term conditions [28, 29]. Regarding future needs, there was a preference for a variety of psychological support interventions including access to cognitive behavioural therapy and increased peer support.

In addition to depression and sleep difficulties, fatigue was identified as one of the most common issues respondents wanted support with. This reinforces past research which identified fatigue as being a highly common and debilitating symptom across the spectrum of advanced kidney disease [30]. There is also emerging evidence in CKD for the potential of behavioural and psychological interventions for fatigue management [31]. Whilst digital interventions have a critical role in accessible delivery for a range of outcomes, notably mental health-related quality of life [19], many people living with CKD require individual specialist psychosocial support and treatment.

In the primary analysis, age (being younger) and female gender were associated with increased symptoms of both depression and anxiety. Current use of mental health medication was associated with lower depression and anxiety compared to individuals not currently taking medication (during previous 12 months). This might be because approximately two thirds had been on medication for over 12 months, and that there remains variability in screening practices for depression and anxiety across kidney services [20]. Receiving talking therapy was associated with higher symptoms of depression and anxiety, reinforcing the need for treatment. It could be argued that this was the result of potentially long waiting lists, since distress commonly increases slightly at the beginning of some therapies. We found no evidence of any centre effects. Whilst there is little literature to make direct comparison in relation to anxiety and depression in CKD, there are contrasting findings in the related area of patient experience of haemodialysis care, where centre effects appear more dominant than person-level factors [32].

This study is the first involving people with CKD to show an association between greater mental health self-efficacy, perceived capability and opportunity for mental health help-seeking, with lower symptoms of depression and anxiety. To our knowledge this is a novel finding in CKD. Capability, Opportunity, and Motivation is predominantly applied to health behaviours and shown to be important in the context of long-term conditions including stroke rehabilitation [33]. Our findings suggest the utility of Capability, Opportunity, and Motivation for explaining variability in depression and anxiety symptoms, which may be in part due to mental health help-seeking behaviours. Interestingly, in a sub-analysis, Capability, Opportunity, and Motivation constructs were unrelated to centre characteristics including psychosocial staff full time equivalent, albeit the numbers were small and therefore difficult to reliably interpret. Mental health self-efficacy has been shown to be associated with improved mental health outcomes [25], whilst self-efficacy more generally is associated with increased psychosocial functioning and better emotional regulation [34]. Our findings suggest that interventions designed to increase mental health self-efficacy, capability and opportunity may help overcome some of the barriers to help-seeking and accessing psychosocial kidney care, though with inevitable resource implications.

This study explored the experiences of CKD patients receiving treatment from 56 different UK kidney centres, capturing the experiences of patients of a range of psychosocial services provided by these centres. The survey, which was developed closely with Patient and Public Involvement members, screened for mental health symptoms in CKD patients and collected information about their history of accessing mental health treatments to provide a more nuanced view of service provision in relation to clinically important mental health outcomes. However, the findings should be interpreted within the context of several limitations. Firstly, it should be noted that given the design, the proportion of individuals meeting screening cut-offs on the PHQ-8 and GAD-7 cannot be used as estimates of prevalence in the wider population. Due to lower-than-expected recruitment for our clinical study, we sought approval to collect a community online sample. An advantage of this was that we were able to draw inference across a larger number of centres than originally planned. However, there were some differences between the online and centre recruited samples. Differences across key demographic and clinical variables were adjusted for in subsequent analyses but ultimately the sample may not be representative due to possible selection and recall bias, given the higher levels of mental health morbidity reported in the online sample. Our study sample also under-represents people who are from ethnic minority backgrounds, which limits the ability to fully capture the needs and experiences of these individuals. Furthermore, our findings may not be generalisable to countries with different healthcare and social care systems.

A key study limitation is the number of participants represented in each of the centre characteristic profiles, limiting the reliability of the case-mixed models which revealed no associations with centre characteristics. We used the size and urban–rural classifications as indicators of psychosocial service provision within centres. This was based on the presumption that larger centres in urban areas might have had greater levels of service provision than smaller centres in rural areas. The failure to observe any link between urban–rural areas and levels of depression and anxiety may partly be due to the complexity of health inequalities between these areas, which are likely better explained by other factors as determined by the social, economic, and cultural circumstances specific to each location. Furthermore, given that all clinical data were self-reported, we cannot verify these aspects. For instance, we did not have specific information regarding aspects of treatments (including medication doses and adjustments), referral times and, for those receiving current talk therapies, how many sessions people had completed, and the complexities of their case presentation. Since all of our sample was under secondary care, we did not capture the experiences of those in primary care, where differences might exist, including those with earlier stages of kidney disease. Lastly, the availability of full time equivalent psychosocial staffing was limited to a smaller number of centres, some of which were purposively selected for recruitment based on location and psychosocial provision. This potentially adds bias and unreliability in this sub-analysis, therefore caution is needed when interpreting these data with a need for larger replication studies.

## Conclusions

Our study is the first to investigate depression and anxiety symptoms across UK kidney centres, finding little evidence of symptom variability. Use of antidepressants—particularly SSRIs, and talking therapies—particularly counselling and cognitive behavioural therapy, are high, yet there likely remains unmet need. Given patient preferences, psychosocial care should, where possible, be administered face to face, and as part of the kidney service. Greater support for fatigue and sleep disorders is also required. Patient-related factors (age and sex) and behavioural characteristics (self-efficacy, capabilities and opportunities) were related to variation of these symptoms. Levels of psychosocial provision showed a weak negative association with depression symptoms. Although our findings should be interpreted in the light of the small sample size and potential for bias, they could be helpful in guiding future research needs and designing service provision.

## Supplementary Information

Below is the link to the electronic supplementary material.

Supplementary file1 (DOCX 87 KB)

## Data Availability

Participants did not consent for their data to be shared.
