# Supplementary material for: Depression and anxiety in people with kidney disease: understanding symptom variability, patient experience and preferences for mental health support
Source: J Nephrol. 2025 Jan 12;38(2):675–86. doi: 10.1007/s40620-024-02194-1 (PMC11961520; doi:10.1007/s40620-024-02194-1)
Supplement: Supplementary file 1 — Supplementary file1 (DOCX 87 KB) [file 40620_2024_2194_MOESM1_ESM.docx]

| Comorbidities | Total | Online | Clinic | Total scores |
| --- | --- | --- | --- | --- |
|  | N=458 | N=261 | N=197 |  |
| Heart Disease |  |  |  |  |
| I have the problem | 49 (10.70%) | 14 (5.36%) | 35 (17.77%) | 128 |
| I receive treatment for it | 50 (10.92%) | 11 (4.21%) | 39 (19.80%) |  |
| It limits my activities | 29 (6.33%) | 13 (4.98%) | 16 (8.12%) |  |
| Previous heart attack |  |  |  |  |
| I have the problem | 20 (4.37%) | 4 (1.53%) | 16 (8.12%) | 45 |
| I receive treatment for it | 17 (3.71%) | 4 (1.53%) | 13 (6.60%) |  |
| It limits my activities | 8 (1.75%) | 0 | 8 (4.06%) |  |
|  |  |  |  |  |
| Diabetes |  |  |  |  |
| I have the problem | 71 (15.50%) | 21 (8.05%) | 50 (25.38%) | 161 |
| I receive treatment for it | 71 (15.50%) | 23 (8.81%) | 48 (24.37%) |  |
| It limits my activities | 19 (4.15%) | 8 (3.07%) | 11 (5.58%) |  |
|  |  |  |  |  |
| Cancer |  |  |  |  |
| I have the problem | 21 (4.59%) | 4 (1.53%) | 17 (8.63%) | 45 |
| I receive treatment for it | 16 (3.49%) | 5 (1.92%) | 11 (5.58%) |  |
| It limits my activities | 8 (1.75%) | 3 (1.15%) | 5 (2.54%) |  |
|  |  |  |  |  |
| Lung disease |  |  |  |  |
| I have the problem | 13 (2.84%) | 4 (1.53%) | 9 (4.57%) | 34 |
| I receive treatment for it | 12 (2.62) | 5 (1.92%) | 7 (3.55) |  |
| It limits my activities | 9 (1.97%) | 3 (1.15%) | 6 (3.05%) |  |
|  |  |  |  |  |
| Arthritis |  |  |  |  |
| I have the problem | 76 (16.59%) | 39 (14.94%) | 37 (18.78%) | 162 |
| I receive treatment for it | 28 (6.11%) | 16 (6.13%) | 12 (6.09%) |  |
| It limits my activities | 58 (12.66%) | 33 (12.64%) | 25 (12.69%) |  |

**Supplementary File**

**Supplementary table1:** Self-reported comorbidity

**Supplementary figure 1:** Self-reported comorbidity

| Variables |  | **Total** | **Online** | **Clinic** | **P-value** |
| --- | --- | --- | --- | --- | --- |
|  |  |  |  |  |  |
| Size classification          Urban-rural classification | 1 Small <700  2 Medium 700-1400  3 Large 1400-2100  4 V Large 2100+    Rural (≤ 251)  Urban (>251) | 102(22.6%)  216 (47.9%)  87 (19.3%)  46 (10.2%)     39 ( 8.6%)  412 (91.4%) | 34 (13.4%)  109 (42.9)  65 (25.6)  46 (18.1)    12 (4.7)  242 (95.3) | 68 (34.5%)  107 (54.3%)  22 (11.2%)  0  27 (13.7%)  170 (86.3%) | <0.001  <0.001 |

**Supplementary table 2:** Patients via Centre profile

**Supplementary table 3:** History of mental health diagnosis

| Mental Health condition | Total | Online | Clinic |
| --- | --- | --- | --- |
| Depression | 259 (56.6%) | 180 (69.0%) | 79 (40.1%) |
| Anxiety | 186 (40.6%) | 141 (54.0%) | 45 (22.8%) |
| Bipolar | 6 (1.31%) | 6 (2.3%) | 0 |
| Post-Natal Depression | 37 (8.1%) | 29 (11.1%) | 8 (4.1%) |
| Seasonal Affective Disorder | 67 (14.6%) | 52 (19.9%) | 15 (7.6%) |
| Other Anxiety Disorder | 82 (17.9%) | 58 (22.2%) | 24 (12.2%) |
| PTSD | 77 (16.8%) | 59 (22.6%) | 18 (9.1%) |
| Adjustment Disorder | 24 (5.2%) | 13 (5.0%) | 11 (5.6%) |
| None Of The Above | 119 (26.0%) | 40 (15.3%) | 79 (40.1%) |

PTSD: Post traumatic stress disorder

**Supplementary table 4:** Health professionals approached to discuss mental health

| HCP | Total | Online | Clinic |
| --- | --- | --- | --- |
| GP | 88 (19.21%) | 67 (25.67%) | 21 (10.66%) |
| Nephrologist | 51 (11.14%) | 44 (16.86%) | 7 (3.55%) |
| Psychiatrist | 7 (1.53%) | 5 (1.92%) | 2 (1.02%) |
| Psychologist | 26 (5.68%) | 22 (8.43%) | 4 (2.03%) |
| Therapist/counsellor | 59 (12.88%) | 46 (17.62%) | 13 (6.60%) |
| Renal nurse | 58 (10.92%) | 38 (14.56%) | 12 (6.09%) |
| Other HCP | 25 (5.46%) | 17 (6.51%) | 8 (4.06%) |
| None of the above | 25 (5.46%) | 21 (8.05%) | 4 (2.03%) |

**Supplementary table 5:** Patterns of medication used for mental health

| *Variable* |  | ***Total*** | **Online** | **Clinic** |  | **P-value** |
| --- | --- | --- | --- | --- | --- | --- |
| Medication used for mental health  When medication was given  Type of medication in the last 12 months  Medication prescribed by  Current use of medication  How often medication is reviewed  Did a doctor stop the medication | No  Yes  Unsure  Last 12 months  Over 12 months ago  Citalopram  Fluoxetine  Sertraline  Mirtazapine  Venlafaxine  Escitalopram  Other  GP  Psychiatrist  Other medical professional  No  Yes  Prefer not to say  Monthly  Every six months  Yearly  Other specified  No  Yes | *N (%)*  126(30)  149(35.5)  3 (0.7)  52(35.6)  94 (64.4)  9 (2)  9 (2)  22 (4.8)  8 (1.7)  5 (1.1)  1 (0.2)  11 (2.4)  45 (83)  7 (13)  2 (4)  40 (33.6)  78 (65.5)  1 (0.8)  9 (12)  19 (25)  18 (23)  31 (40)  29 (74)  10 (26) | 123(53)  106(45.7)  3(1.3)  39(37.1)  66(62.9)  7(2.7)  5(1.9)  21(8)  7(2.7)  4(1.5)  1(0.4)  6(2.3)  35(81)  6(14)  2(5)  40(37)  67(62)  1(0.9)  7(11)  17(26)  17(26)  25(38)  29(74)  10(26) | 3(1.6)  43(22.9)  0  13(31.7)  28(68.3)  2(1)  4(2)  1(0.5)  1(0.5)  1(0.5)  0  5(2.5)  10(91)  1(9)  0  0  11(100)  0  2(18)  2(18)  1(9)  6(54)  0  0 |  | <0.001  0.54  <0.001  0.68  0.041  0.094 |

**Supplementary table 6:** Patterns of talk therapies used

| *Variable* |  | *Total* | *Online* | *Clinic* |
| --- | --- | --- | --- | --- |
| Received counselling/ therapy in the last 24 months  Type of psychological support in the last 12 months  Provided by  Mode of delivery  Number and duration of sessions  Complementary therapies used | No  Yes  CBT  Counselling  ACT  Other specified  IAPT  Renal service  Private provider  Other  Online therapist-supported  One to one  Group therapy  Other  <6 30-60 min  >7 lasting 60 min  Acupressure or acupuncture  Aromatherapy  Mindfulness  None  Other | 302 (72.1%)  117 (27.9%)  48 (10.5%)  65 (14.2%)  10 (2.2%)  24 (5.2%)  *38 (14.6)*  *23(8.8)*  *16(6.1)*  *16(6.1)*  6 (7)  74 (85%)  1 ( 1%)  6 ( 7%)  37 (43%)  50 (57%)  7 ( 1.5%)  12 ( 2.6%)  69 (15.1%)  314 (68.6%)  33 ( 7.3%) | *147 (63.4%)*  *85 (36.6%)*  *37 (14.2%)*  *47 (18.0%)*  *8 ( 3.1%)*  *18 ( 6.9%)*  *38 (14.6)*  *23(8.8)*  *16(6.1)*  *16(6.1)*  *6 ( 8%)*  *67 (84%)*  *1 ( 1%)*  *6 ( 8%)*  *35 (44%)*  *45 (56%)*  *3 ( 1.1%)*  *9 ( 3.4%)*  *50 (19.2%)*  *159 (60.9%)*  *25 ( 9.6%)* | *155 (82.9%)*  *32 (17.1%)*  *11 ( 5.6%)*  *18 ( 9.1%)*  *2 ( 1.0%)*  *6 ( 3.0%)*  *Not available*  *0*  *7 (10)*  *0*  *0*  *2 (29)*  *5 (71)*  *4 ( 2.0%)*  *3 ( 1.5%)*  *19 ( 9.6%)*  *155 (78.7%)*  *8 ( 4.1%)* |

**Supplementary table 7:** Preference for psychological support

| *Psychological support* | *Preference* | | ***Total*** | *Online* | *Clinic* |
| --- | --- | --- | --- | --- | --- |
| Provider  Type  Mode  Area for support to be focussed on  Delivered by whom  Timing/ availability | A health care professional who works as part of the renal team  A health care professional who is separate from the renal team  Don't mind  Talking to other people with CKD  Relaxation therapy  Mindfulness techniques  Self-management support  Stress management  Increasing Access to Psychological Therapies (IAPT)  Counselling  Cognitive Behavioural Therapy (CBT)  Psychotherapy or psychoanalysis  Clinical psychology service  Complementary therapy  Couples or family therapy  Other, please state  Face-to-face individual  Face-to-face group  Telephone individual  Telephone group  Video call tool e.g. Zoom  App or website-based self-help  App or website-based guided by a healthcare  Email  Written materials e.g. leaflets  Fatigue  Sleep  Low mood  Depression  Anxiety  Fear  Anger and frustration  Guilt  Self-confidence  Body image  Work  Exercising  Planning for the future  Isolation and loneliness  My role and identity  Eating a healthy diet  Sexual difficulties  Relationship difficulties  Social life  Taking my medication  Family planning  Friendships  Stopping smoking  Stopping or reducing alcohol intake  Within your nephrology team e.g. Nephrology Nurse Specialist; Nephrologist; Renal social worker  Mental Health specialist e.g. Counsellor or Psychotherapist; Psychologist; Psychiatrist; Mental Health Nurse  Family/Friends/Partner support  Another person with CKD  GP Practice e.g. Nurse, GP or pharmacist  Allied health professional e.g. Occupational Therapist; Dietician; Physiotherapist Complementary Therapist  Kidney disease charity  Mental health charity  Faith-based organisation  Only when it is needed  Continuously (i.e. part into regular care)  At diagnosis of CKD  During discussion of a change of treatment e.g. starting dialysis  Within 6 months of diagnosis of CKD  Within 6-12 months of diagnosis of CKD  Within 12-18 months of diagnosis of CKD  Not at all | 164(40.7%)  50 (12.4%)  189(46.9%)  192(41.9%)  147(32.1%)  117(25.5%)  157(34.3%)  112(24.5%)  76 (16.6%)  200(43.7%)  87 (19.0%)  54 (11.8%)  68 (14.8%)  91 (19.9%)  48 (10.5%)  24 ( 5.2%)  331(86.4%)  *84 (34.9%)*  *104(39.7%)*  13 ( 6.5%)  95 (36.0%)  61 (26.9%)  60 (25.9%)  53 (24.2%)  64 (28.7%)  218(67.9%)  172(59.3%)  199(60.9%)  150(51.0%)  199(60.3%)  129(44.9%)  103(38.7%)  75 (29.4%)  118(41.8%)  129(44.5%)  53 (23.3%)  104(39.8%)  107(40.2%)  84 (32.3%)  63 (25.5%)  111(42.5%)  54 (23.6%)  50 (21.2%)  67 (27.9%)  26 (12.4%)  21 (10.0%)  40 (17.9%)  11 ( 5.6%)  4 ( 2.0%)  249(74.1%)  208(63.4%)  113(44.8%)  109(41.1%)  71 (31.6%)  71 (30.1%)  55 (24.3%)  116(43.0%)  62 (25.9%)  12 ( 6.1%)  230(79.3%)  137(45.7%)  83 (33.1%)  107(40.4%)  33 (15.3%)  9 ( 4.5%)  7 ( 3.5%)  8 ( 4.1%) | | *97 (44.5%)*  *32 (14.7%)*  *89 (40.8%)*  *110(42.1%)*  *88 (33.7%)*  *75 (28.7%)*  *92 (35.2%)*  *61 (23.4%)*  *59 (22.6%)*  *127(48.7%)*  *54 (20.7%)*  *43 (16.5%)*  *45 (17.2%)*  *55 (21.1%)*  *29 (11.1%)*  *17 ( 6.5%)*  *189(100.0%)*  *47 (100.0%)*  *68 (100.0%)*  *7 (100.0%)*  *70 (100.0%)*  *33 (100.0%)*  *38 (100.0%)*  *25 (100.0%)*  *29 (100.0%)*  *127(100.0%)*  *96 (100.0%)*  *133(100.0%)*  *100(100.0%)*  *136(100.0%)*  *93 (100.0%)*  *72 (100.0%)*  *61 (100.0%)*  *88 (100.0%)*  *96 (100.0%)*  *33 (100.0%)*  *67 (100.0%)*  *72 (100.0%)*  *66 (100.0%)*  *53 (100.0%)*  *67 (100.0%)*  *35 (100.0%)*  *42 (100.0%)*  *46 (100.0%)*  *15 (100.0%)*  *17 (100.0%)*  *29 (100.0%)*  *4 (100.0%)*  *2 (100.0%)*  *142(100.0%)*  *134*  *58*  *71*  *31*  *42*  *32*  *76*  *45*  *4*  *96*  *106*  *57*  *71*  *22*  *8*  *6*  *2* | *67 (36.2%)*  *18 ( 9.7%)*  *100(54.1%)*  *82 (41.6%)*  *59 (29.9%)*  *42 (21.3%)*  *65 (33.0%)*  *51 (25.9%)*  *17 ( 8.6%)*  *73 (37.1%)*  *33 (16.8%)*  *11 ( 5.6%)*  *23 (11.7%)*  *36 (18.3%)*  *19 ( 9.6%)*  *7 ( 3.6%)*  *142(73.2%)*  *37 (19.1%)*  *36 (18.6%)*  *6 ( 3.1%)*  *25 (12.9%)*  *28 (14.4%)*  *22 (11.3%)*  *28 (14.4%)*  *35 (18.0%)*  *91 (46.9%)*  *76 (39.2%)*  *66 (34.0%)*  *50 (25.8%)*  *63 (32.5%)*  *36 (18.6%)*  *31 (16.0%)*  *14 ( 7.2%)*  *30 (15.5%)*  *33 (17.0%)*  *20 (10.3%)*  *37 (19.1%)*  *35 (18.0%)*  *18 ( 9.3%)*  *10 ( 5.2%)*  *44 (22.7%)*  *19 ( 9.8%)*  *8 ( 4.1%)*  *21 (10.8%)*  *11 ( 5.7%)*  *4 ( 2.1%)*  *11 ( 5.7%)*  *7 ( 3.6%)*  *2 ( 1.0%)*  *107(55.2%)*  *74(38.1%)*  *55(28.4%)*  *38(19.6%)*  *40(20.6%)*  *29(14.9%)*  *23(11.9%)*  *40(20.6%)*  *17( 8.8%)*  *8( 4.1%)*  *134(69.1%)*  *31 (16.0%)*  *26 (13.4%)*  *36 (18.6%)*  *11 ( 5.7%)*  *1 ( 0.5%)*  *1 ( 0.5%)*  *6 ( 3.1%)* |

**Supplementary table 8:** Summary of depression (PHQ-8) and anxiety (GAD-7) scores

| *Variable* | *Cronbach’s α* | *Mean* | *SD* | *95% CI* |
| --- | --- | --- | --- | --- |
| **Anxiety Total**  **Depression Total** | .89  .89 | 6.0  7.9 | .25  .29 | 5.57 – 6.58  7.36 – 8.52 |
| **Anxiety Online**  **Depression Online** | .89  .89 | 7.3  9.3 | .34  .39 | 6.68 - 8.04  8.61 - 10.15 |
| **Anxiety Clinic**  **Depression Clinic** | .89  .89 | 4.3  6.0 | .34  .41 | 3.67 – 5.05  5.26 – 6.89 |

*α: Cronbach’s alpha; SD: standard deviation; CI: Confidence Interval*

**Supplementary table 9:** Psychological correlates of depression and anxiety symptoms.

|  |  | 1 | 2 | 3 | 4 | 5 |
| --- | --- | --- | --- | --- | --- | --- |
| 1 | Depression | - |  |  |  |  |
| 2 | Anxiety | 0.81 | - |  |  |  |
| 3 | Capability | -0.50 | -0.42 | - |  |  |
| 4 | Opportunity | -0.43 | -0.38 | 0.61 | - |  |
| 5 | Motivation | -0.55 | -0.45 | 0.66 | 0.63 | - |
| 6 | Mental Health Self-efficacy | -0.76 | -0.72 | 0.61 | 0.51 | 0.66 |

*All correlations p<0.01*
